# Primary malignant melanoma of the breast: A case report and review of the literature

**DOI:** 10.3892/ol.2014.2120

**Published:** 2014-05-07

**Authors:** YUJUN HE, JIANGHONG MOU, DONGLIN LUO, BO GAO, YAYUAN WEN

**Affiliations:** 1 Department of General Surgery, Daping Hospital and Research Institute of Surgery, Third Military Medical University, Chongqing 400042, P.R. China; 2 Department of Pathology, Daping Hospital and Research Institute of Surgery, Third Military Medical University, Chongqing 400042, P.R. China

**Keywords:** breast, primary malignant melanoma, diagnosis, therapy

## Abstract

Malignant melanoma predominantly occurs in the skin and mucous membranes, thus, malignant melanoma of the breast is particularly rare. In the current study, a case of a 26-year-old female with a malignant melanoma of the breast is presented. On diagnosis of the patient, extensive metastasis had occurred. The patient refused any treatment and succumbed two months after the initial diagnosis. The prognosis for patients with this rare tumour of the breast is somewhat poor. Early diagnosis, correct surgical resection and comprehensive adjuvant therapy are the key procedures that may improve the patient survival rate. The current case report aims to increase the awareness of uncommon tumours of the breast.

## Introduction

Malignant melanoma predominantly occurs in the skin, mucous membranes and the choroid. Malignant melanoma of the breast is particularly rare. The incidence of primary melanoma of the breast is <5% of all melanomas (1). Observation of the clinical pathological features, immunohistochemical staining methods and tissue tissue origin are required to identify primary malignant melanoma of the breast, as well as other types of breast tumour. Surgical resection is the commonly adopted treatment method for malignant melanoma, supplemented by chemo-, radio- and immunotherapy treatments resulting in a comprehensive treatment strategy.

In the current study, a 26-year-old female patient exhibiting a primary malignant melanoma of the breast is presented, and the clinical and pathological features, diagnosis and treatments are discussed in correlation with the literature. Patient provided written informed consent.

## Case report

**Figure 1:**
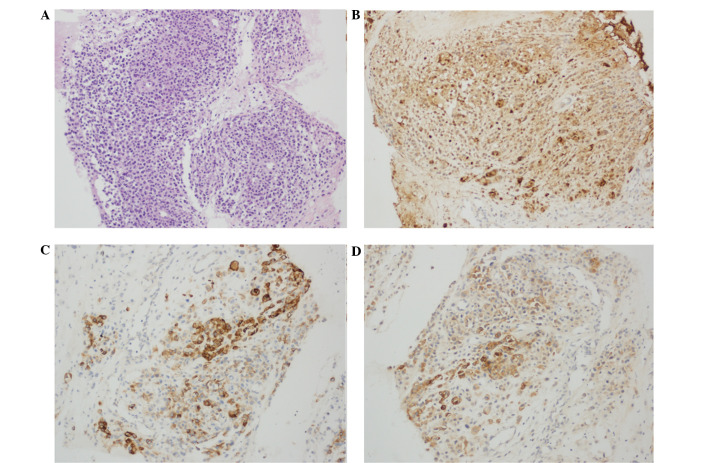
Pathological morphology features and immunohistochemistry results of the patient. (A) Hematoxylin and eosin-stained section shows tumour cell pleomorphism and nuclear atypia. Immunohistochemical staining in the tumour cells for (B) S-100, showing nuclear and cytoplasmic positivity (streptavidin-peroxidase staining); (C) HMB-45, showing strong cytoplasmic positivity (streptavidin-peroxidase staining); and (D) melan-A, showing cytoplasmic positivy (streptavidin-peroxidase staining). Magnification, ×100.

A 26-year-old female was admitted to the Department of General Surgery of Daping Hospital and Research Institute of Surgery (Chongqing, China), due to the presence of a painless mass in the left breast for three months. The patient indicated that the mass had recently grown rapidly. The patient had no notable medical history or family history of carcinoma. Clinical examination revealed a 3×2-cm firm irregular mass in the upper inner quadrant of the left breast. There was no change in the appearance of the local skin, no discharge from, or retraction of, the nipple. A small number of lymph nodes were palpated in the left axilla. The breast magnetic resonance imaging result indicated left breast cancer due to the presence of enlarged left axillary lymph nodes. A chest computed tomography (CT) scan demonstrated widespread lung and pleural nodules, indicating lung and pleural metastases. An emission CT whole body bone scintigraphy indicated destruction to multiple ribs, the cervical vertebrae and thoracic bone. A core needle biopsy of the breast mass and hematoxylin and eosin staining demonstrated that the mass tissue was comprised of a large distribution of diffuse small cells. Those cells were round or oval, with large nuclei and nucleoli, and abundant cytoplasm. No significant intracellular pigmentation was observed (Fig. 1A). Immunohistochemistry demonstrated that the tumour cells were immunopositive for S-100, HMB-45 and melan-A (Fig. 1B–D). However. a panel of markers that included epithelial markers, such as cytokeratin (CK) and epithelial membrane antigen (EMA), and mesenchymal markers, such as vimentin, smooth muscle antigen (SMA), estrogen receptor, progesterone receptor and HER2 were negative. The percentage of Ki-67-positive cells was 30%.

Based on the pathologic and immunohistochemical features, a diagnosis of malignant melanoma was proposed. Careful examination of the skin and mucous membranes failed to reveal a malignant melanoma. Therefore this patient was diagnosed with a primary malignant melanoma of the left breast with extensive metastasis. The patient refused surgery and further treatment and was automatically discharged. Two months later, the patient succumbed as a result of widespread metastases.

## Discussion

Malignant melanoma is a highly malignant tumour that is derived from melanocytes. The incidence of malignant melanoma has risen markedly over the last decade. It occurs anywhere on the body, however, is commonly found in the skin, mucous membranes and the choroid. Primary melanoma of the breast is particularly rare, with an incidence of <5% of all malignant melanomas (1,2). The aetiology of malignant melanoma remains unknown. It is generally hypothesised to be associated with excessive exposure to ultraviolet radiation from the sun. In addition, it is associated with ethnicity, the endocrine and immune systems, chronic stimulation and improper surgery may cause the progression of nevus into malignant melanoma.

Malignant melanoma of the breast has four predominant manifestations: i) Primary malignant melanoma of the breast skin; ii) malignant melanoma metastasis to the breast; iii) in-transit metastases to breast tissue and skin; and iv) primary malignant melanoma of the breast gland (1). The diagnosis of primary malignant melanoma of the breast is highly dependent on pathological morphology, immunohistochemistry and electron microscopy, amongst other diagnostic techniques, and the following should be noted during diagnosis: i) Pleomorphism of tumour cells and nuclear atypia; ii) scattered intracellular pigment granules (although there are 6–10% of malignant melanomas exhibiting little or no pigment, which are termed amelanotic melanoma) (3); iii) immunohistochemistry results demonstrating positive expression of the proteins S-100, HMB-45 and melan-A (4); iv) electron microscopy identifying melanosome and former melanosome (5); v) the edge of the tumour tissue and normal breast tissue does not exhibit a transition; and vi) exclusion of tumour metastases and tumor invasion from neighboring sites. The diagnosis of malignant melanoma is occasionally particularly complex and, therefore, requires the use of immunohistochemical staining for its identification. Positive expression of S-100 is an exceptionally sensitive indicator for malignant melanoma, however, it is also expressed in 50% of breast cancer cases. Therefore it must be observed in combination with a positive expression of HMB-45 and melan-A for the diagnosis of primary melanoma of the breast. Whereas other indicators, such as CK, vimentin and SMA, demonstrate a negative expression and signal the presentation of other types of tumours. Furthermore, Ki-67 staining may be used to distinguish between benign and malignant tumours (6,7).

In the present study, the first symptom that was noted by the patient was the tumour in the left breast. Based on the clinical examination, histopathological features and results from immunohistochemical staining, the patient was diagnosed with a primary malignant melanoma of the breast.

The treatment of primary malignant melanoma of the breast is the same as that for other malignant melanoma located elsewhere on the body. The primary treatment method is surgical resection, with an appropriate combination of chemo-, radio-, immuno- and targeted therapy (1,8). Wide local excision is the predominant surgical approach. It is generally hypothesised that a cutting edge of 2 cm ensures the reliability of the surgery. A mastectomy does not improve the patient’s prognosis, and a comprehensive axillary lymph node dissection is required when preoperative axillary lymph node metastasis is identified and confirmed. A sentinel lymph node biopsy reduces the requirement for an unnecessary lymph node dissection (9,10).

The role of adjuvant chemo- or radiotherapy, either singularly or in combination, remains unknown with regard to their efficacy in malignant melanoma. Chemotherapy is commonly used for pre- and postoperative adjuvant therapy and for those who are not suitable for, or refuse, surgery or for those patients who exhibit widespread metastases. The chemotherapy programme is usually with a dacarbazine-based treatment plan, however, the effective rate is only 7–13% (11). Other commonly used agents include temozolomide, cisplatin and taxol; multi-agent chemotherapy may improve the treatment outcome. Adjuvant radiotherapy may be performed when removal of the lesion is not possible, there are positive margins, the lymph node size is >3 cm, the number of lymph nodes involved exceeds four or when there is local recurrence or distant metastases. Although adjuvant radiotherapy decreases the local regional failure from 30 to 10%, the survival rate of patients remains unknown (12,13).

Malignant melanoma is an immunogenic tumour and adjuvant immunotherapy is associated with high-risk tumours. Interferon, interleukin-2 and other biological response modifiers for malignant melanoma have a moderate effect. It is reported that immunotherapy in combination with chemotherapy may improve the efficiency of patient treatment, however, the long-term effects require further investigation (14–16).

In recent years, numerous novel biological and molecular targeted therapies have been adopted for the treatment of malignant melanoma. Clinical studies have identified that ipilimumab (a monoclonal antibody that blocks cytotoxic T lymphocyte-associated antigen 4) and vemurafenib (oncogenic BRAF-inhibitor agent) improve the overall response rate, and prolong the progression-free survival and overall survival for advanced malignant melanoma patients. However, the specific efficacy is currently being investigated and evaluated (17–19).

In conclusion, primary malignant melanoma of the breast is a particularly rare type of tumour and the prognosis is somewhat poor. The diagnosis depends on histopathological assessment and immunohistochemical staining combined with a detailed clinical history and careful physical examination. Early diagnosis, correct surgical resection and comprehensive adjuvant therapy are significant factors for improving the patient survival rate.

## References

[1] Kurul S, Taş F, Büyükbabani N (2005). Different manifestations of malignant melanoma in the breast: a report of 12 cases and a review of the literature. Jpn J Clin Oncol.

[2] Alzaraa A, Sharma N (2008). Primary cutaneous melanoma of the breast: A case report. Cases J.

[3] Duggal R, Srinivasan R (2010). Primary amelanotic melanoma of the cervix: case report with review of literature. J Gynecol Oncol.

[4] Bonetti F, Pea M, Martignoni G (1991). False-positive immunostaining of normal epithelia and carcinomas with ascites fluid preparations of antimelanoma monoclonal antibody HMB45. Am J Clin Pathol.

[5] Taatjes DJ, Arendash-Durand B, von Turkovich M, Trainer TD (1993). HMB-45 antibody demonstrates melanosome specificity by immunoelectron microscopy. Arch Pathol Lab Med.

[6] Ohsie SJ, Sarantopoulos GP, Cochran AJ, Binder SW (2008). Immunohistochemical characteristics of melanoma. J Cutan Pathol.

[7] Lee AH (2013). Use of immunohistochemistry in the diagnosis of problematic breast lesions. J Clin Pathol.

[8] Davar D, Tarhini AA, Kirkwood JM (2012). Adjuvant therapy for melanoma. Cancer J.

[9] Biswas A, Goyal S, Jain A (2014). Primary amelanotic melanoma of the breast: combating a rare cancer. Breast Cancer.

[10] Thompson JF, McCarthy WH, Bosch CM (1995). Sentinel lymph node status as an indicator of the presence of metastatic melanoma in regional lymph nodes. Melanoma Res.

[11] Avril MF, Aamdal S, Grob JJ (2004). Fotemustine compared with dacarbazine in patients with disseminated malignant melanoma: a phase III study. J Clin Oncol.

[12] Lee RJ, Gibbs JF, Proulx GM (2000). Nodal basin recurrence following lymph node dissection for melanoma: implications for adjuvant radiotherapy. Int J Radiat Oncol Biol Phys.

[13] Calabro A, Singletary SE, Balch CM (1989). Patterns of relapse in 1001 consecutive patients with melanoma nodal metastases. Arch Surg.

[14] Thompson JF, Scolyer RA, Kefford RF (2005). Cutaneous melanoma. Lancet.

[15] Verma S, Quirt I, McCready D (2006). Systematic review of systemic adjuvant therapy for patients at high risk for recurrent melanoma. Cancer.

[16] Hauschild A, Weichenthal M, Rass K (2010). Efficacy of low-dose interferon α2a 18 versus 60 months of treatment in patients with primary melanoma of >= 1.5 mm tumor thickness: results of a randomized phase III DeCOG trial. J Clin Oncol.

[17] Chapman PB, Hauschild A, Robert C, BRIM-3 Study Group (2011). Improved survival with vemurafenib in melanoma with BRAF V600E mutation. N Engl J Med.

[18] Ribas A, Hersey P, Middleton MR (2012). New challenges in endpoints for drug development in advanced melanoma. Clin Cancer Res.

[19] Spagnolo F, Queirolo P (2012). Upcoming strategies for the treatment of metastatic melanoma. Arch Dermatol Res.

